# Plasma Asprosin Concentrations Are Increased in Individuals with Glucose Dysregulation and Correlated with Insulin Resistance and First-Phase Insulin Secretion

**DOI:** 10.1155/2018/9471583

**Published:** 2018-03-20

**Authors:** Yuren Wang, Hua Qu, Xin Xiong, Yuyang Qiu, Yong Liao, Yingchun Chen, Yi Zheng, Hongting Zheng

**Affiliations:** ^1^Department of Endocrinology, Translational Research Key Laboratory for Diabetes, Xinqiao Hospital, Third Military Medical University, Chongqing, China; ^2^Department of Endocrinology, Armed Police Hospital of Chongqing, Chongqing, China; ^3^Department of Medicinal Chemistry, College of Pharmacy, Third Military Medical University, Chongqing, China

## Abstract

**Background:**

Adipokines are reported to participate in many common pathologic processes of glucose dysregulation, such as insulin resistance, *β*-cell dysfunction, and chronic inflammation.

**Objective:**

To detect the concentrations of plasma asprosin in subjects with impaired glucose regulation (IGR) and newly diagnosed type 2 diabetes (nT2DM) and its relationship to parameters of glucose and lipid metabolism, insulin resistance, and pancreatic *β*-cell function.

**Methods:**

143 eligible participants were included and were divided into three groups including normal glucose regulation (NGR, *n* = 52), IGR (*n* = 40), and nT2DM group (*n* = 51). The intravenous glucose tolerance test (IVGTT) and clinical and biochemical parameters were measured in all participants.

**Results:**

Plasma asprosin levels were higher in IGR (82.40 ± 91.06 ng/mL, *P* < 0.001) and nT2DM (73.25 ± 91.69 ng/mL, *P* < 0.001) groups compared with those in the NGR (16.22 ± 9.27 ng/mL) group, especially in IGR subjects. Correlation analysis showed that plasma asprosin levels were positively correlated with waist circumference (Wc), fasting plasma glucose (FPG), postchallenge plasma glucose (2hPG), HbA1c, triglyceride (TG), and homeostasis model assessment for insulin resistance (HOMA-IR) and negatively correlated with homeostasis model assessment for *β*-cell function (HOMA-*β*), area under the curve of the first-phase (0–10 min) insulin secretion (AUC), acute insulin response (AIR), and glucose disposition index (GDI) (all *P* < 0.05). Multiple logistical regression analyses revealed that plasma asprosin concentrations were significantly correlated with IGR and nT2DM after controlling for age, sex, BMI, and WHR.

**Conclusions:**

Circulating asprosin might be a predictor of early diagnosis in DM and might be a potential therapeutic target for prediabetes and T2DM.

## 1. Introduction

Diabetes mellitus (DM) has become a severe public health problem globally, as every 6 seconds a person dies from diabetes [1]. Impaired glucose regulation (IGR), including both impaired glucose tolerance (IGT) and impaired fasting glycaemia (IFG) [2], has a high risk to develop as DM [3, 4] and shared many common pathologic mechanisms with DM, such as insulin resistance, *β*-cell dysfunction, and chronic inflammation.

The adipose tissue is not only a place to store fat but also considered as an endocrine organ secreting adipokines, which participate in the pathologic processes of DM and IGR. Perturbations in these adipokines can cause pathological alteration in glucose metabolism, often with severe consequences. Asprosin, a novel adipokine found by Romere et al. in 2016, is the C-terminal cleavage product of profibrillin (encoded by *FBN1*) [5]. It is secreted by the white adipose tissue and targets the liver to fasting-responsive increase plasma glucose and insulin levels [5]. Previous study showed a pathological elevation of asprosin in human and mice with insulin resistance, and its loss of function via immunologic or genetic methods has a profound glucose- and insulin-lowering effect in mice [5]. In addition, asprosin was reported to activate the G protein-cAMP-PKA axis in the liver [5], which has been demonstrated to have an amelioration effect on chronic inflammation [6, 7]. All these results suggested asprosin may play a role in glucose metabolism, whereas no direct data are available for the role of circulating asprosin in type 2 diabetes (T2DM) and IGR subjects. Therefore, we conducted a cross-sectional study to evaluate the plasma asprosin concentrations in normal glucose regulation (NGR), newly diagnosed T2DM (nT2DM), and IGR subjects and analyze its correlation with metabolic parameters and inflammation.

## 2. Methods

### 2.1. Study Subjects

One hundred and forty-three Chinese subjects aged 27 to 75 years were recruited in our study. According to the diagnostic criteria of WHO in 1998, that is, for DM, fasting plasma glucose (FPG) value ≥ 7.0 mmol/L or 2 h postglucose challenge (2hPG) ≥ 11.1 mmol/L or both; for IGR, FPG ≥ 6.1 mmol/L and ≤7.0 mmol/L and 2hPG < 7.8 mmol/L (IFG) or FPG < 7.0 mmol/L and 2hPG ≥ 7.8 mmol/L and ≤11.1 mmol/L (IGT) [2], participants were divided into three groups: NGR (*n* = 52), IGR (*n* = 40), and nT2D (*n* = 51) group. The exclusion criteria include the following: (a) subjects with smoking and drinking history; (b) subjects suffering from acute and chronic complications of diabetes; (c) subjects having been diagnosed with IGR or any type of diabetes; (d) subjects having acute and chronic inflammatory diseases; (e) subjects with hepatic or renal disease, systemic corticosteroid treatment, sustained hypertension, or cardiovascular disease; and (f) women who were currently pregnant. The sample size was calculated using PASS software (version 15.0; NCSS, Silver Spring, Md), both the means of groups and the variance were set according to the results of our previous study [8]. The power was set at 0.8, and the significance level was set at 0.05. All experimental protocols were approved by the Ethics Committee of Xinqiao Hospital, Third Military Medical University and registered online (Clinical trial register number ChiCTR-ROC-17010719).

### 2.2. Clinical and Biochemical Evaluations

The height, body weight, waist circumference (Wc), hip circumference, and blood pressure were measured using standard protocols in all subjects. Peripheral venous blood samples were collected in the morning after an 8-hour overnight fasting. Plasma samples were obtained by centrifugation at 1000*g* for 15 minutes at 4°C and were kept at −80°C until used, all within a 3-month period. FPG was assayed using the glucose oxidase method. Glycated hemoglobin (HbA1c) was determined by high-performance liquid chromatography (VARIANTTM II and D-10TM Systems, Bio-Rad, USA). Fasting insulin (FINS) was measured in serum by RIA using human insulin as standard (Linco, St. Charles, MO, USA). Lipid profiles, hypersensitive C-reactive protein (hsCRP), and liver and kidney functions were detected by biochemical autoanalyzer (Beckman CX-7 Biochemical Autoanalyser, Brea, CA, USA). IVGTT studies were performed after an overnight fast; subjects were injected 0.3 g/kg glucose (50%) rapidly within 3 min, then blood samples were taken at 0, 3, 5, 8, 10, 30, 60, and 120 min to measure glucose and insulin levels. The body mass index (BMI) was calculated as the ratio of the weight and squared height. The waist-hip ratio (WHR) was calculated as the ratio of the waist circumferences and hip circumferences. The area under the curve of the first-phase (0 to 10 min) insulin secretion (AUC) was computed by irregular trapezoid area. The acute insulin response (AIR) = (I_3 min_ + I_5 min_)/2. The glucose disposition index (GDI) = log_10_^(AIR × FPG/FINS)^. The homeostasis model assessment of insulin resistance (HOMA-IR) = FPG × FINS/22.5. The homeostasis model assessment of *β* (HOMA-*β*) = 20 × FINS/(FPG − 3.5).

### 2.3. Assessment of Plasma Asprosin Levels

Plasma asprosin levels were determined by a commercial enzyme-linked immunosorbent kit according to the manufacturers' instructions (Human ELISA kit, Wuhan EIAab Science Co. Ltd., China). The intra-assay coefficient of variation was 10%, and the interassay coefficient of variation was 12%. All the assays were performed in duplicate and repeated if there was a > 15% difference between duplicates. No significant cross-reactivity or interference was observed.

### 2.4. Statistical Analyses

All statistical analyses were conducted by SPSS software (IBM, Armonk, NY, version 19.0). Data were presented as mean values ± standard deviation (SD). Normal distribution of the data was detected using Kolmogorox-Smirnov test. Several variables showed skewed distribution and were logarithmically transformed into normal distribution before statistical analysis. Analysis of variance (ANOVA) was performed for group comparisons. Interrelationships between variables were estimated using Spearman correlation coefficient. Multivariate logistic regression analyses were used to analyze the association between plasma asprosin and diabetes. *P* values < 0.05 were regarded as statistically significant.

## 3. Results

### 3.1. The Clinical Characteristics

The main clinical characteristics of the subjects in different groups were shown in Table 1. There were no significant differences in serum asprosin levels between men and women (57.64 ± 82.24 versus 53.27 ± 76.08 ng/mL, *P* = 0.744). And there were no significant differences in sex, age, and TC among the three groups (For IGR, *P* = 0.641, *P* = 0.745, and *P* = 0.144, respectively; for nT2DM, *P* = 0.038, *P* = 0.633, and *P* = 0.061, resp.). In comparison to the NGR group, participants with IGR and nT2DM had a higher levels of Wc, FPG, 2hPG, HOMA-IR, TG, and LDL-C (For IGR, *P* < 0.001, *P* < 0.001, *P* = 0.001, *P* = 0.005, *P* < 0.001, and *P* = 0.044, resp.; for nT2DM, *P* < 0.001, *P* < 0.001, *P* < 0.001, *P* < 0.001, *P* = 0.001, and *P* = 0.037, resp.), whereas the levels of HOMA-*β*, AUC, AIR, GDI, and HDL-C were significantly lower in IGR and nT2DM groups (for IGR, *P* < 0.001, *P* = 0.008, *P* = 0.004, *P* = 0.027, and *P* = 0.015, resp.; for nT2DM, *P* < 0.001, *P* < 0.001, *P* < 0.001, *P* < 0.001, and *P* = 0.002, resp.). Besides, compared with the NGR group, higher levels of BMI, WHR, SBP, DBP, HbA1c, FINS, and hsCRP were significant in nT2DM group but not in IGR group (*P* = 0.004, *P* < 0.001, *P* = 0.035, *P* = 0.070, *P* < 0.001, *P* = 0.003, and *P* = 0.032, resp.).

### 3.2. Plasma Asprosin Levels in Different Groups

**Figure 1:**
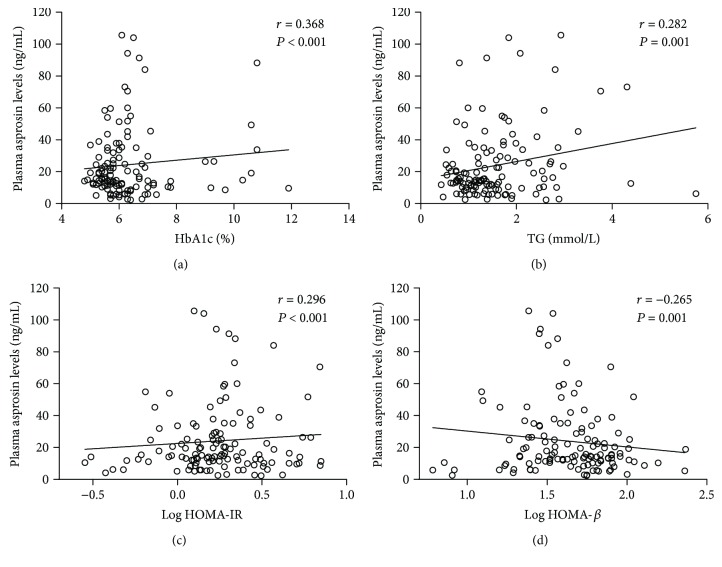
Scatter plots showing the correlation of plasma asprosin levels with HbA1c, TG, HOMA-IR, and HOMA-*β* in all subjects. (a) The plasma asprosin levels positively correlated with HbA1c. (b) The plasma asprosin levels positively correlated with TG. (c) The plasma asprosin levels positively correlated with HOMA-IR. (d) The plasma PGRN levels negatively correlated with HOMA-*β*. TG: triglyceride; HOMA-IR: homeostasis model assessment for insulin resistance; HOMA-*β*: homeostasis model assessment for beta-cell function.

Compared to the NGR group, subjects displayed a significant increased trend of plasma asprosin concentrations both in the IGR and nT2DM group (Table 1, both *P* < 0.001). Interestingly, between the IGR and nT2DM groups, the plasma asprosin concentrations showed an increasing trend in the IGR group (*P* = 0.555), suggesting a correlation between plasma asprosin and glucose dysregulation. To further investigate, we explored the association between asprosin and metabolic parameters. As shown in Table 2, the plasma asprosin levels were positively associated with parameters including Wc, FPG, 2hPG, HbA1c (Figure 1(a)), TG (Figure 1(b)), and HOMA-IR (Figure 1(c)) (*P* = 0.027, *P* < 0.001, *P* = 0.003, *P* < 0.001, *P* = 0.001, and *P* < 0.001, resp.) and were negatively correlated with HOMA-*β* (Figure 1(d)), AUC, AIR, and GDI (*P* = 0.001, *P* < 0.001, *P* < 0.001, and *P* < 0.001, resp.). All these correlations remained statistically significant after adjustment by age.

### 3.3. The First Phase of Glucose-Stimulated Asprosin Secretion

**Figure 2:**
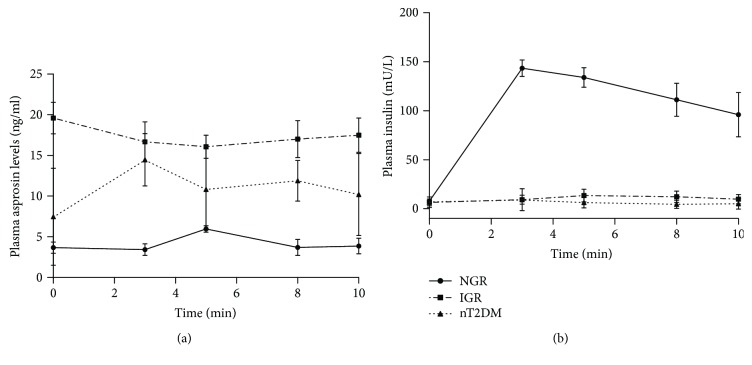
The first phase of glucose-stimulated (a) insulin and (b) asprosin concentrations during IVGTT in healthy, IGR, and T2DM subjects. NGT: normal glucose tolerance; IGR: impaired glucose regulation; T2DM: type 2 diabetes mellitus.

As we know, the impairment of first-phase insulin secretion is a common characteristic in IGR and T2DM; thus, we examined the effects of IVGTT on plasma asprosin levels in the first phase and compared its secretion pattern with insulin. Among the three groups, the plasma asprosin level in the NGR group was the lowest one at every point during the first phase (Figure 2(a)), which was opposite with the trend of insulin secretion (Figure 2(b)). Interestingly, the area under curve of the first-phase asprosin secretion in the IGR group is higher than that in the nT2DM group (171.3 ± 11.75 versus 114.3 ± 15.13, *P* = 0.041, Figure 2(a)), which was consistent with the fasting plasma asprosin concentration we observed but inconsistent with first-phase insulin secretion. However, at least, all these results indicated that the secretion pattern of circulating asprosin levels was dynamically changed with circulating glucose and insulin, suggesting a pronounced association of plasma asprosin with insulin resistance and *β*-cell functions. In light of this and in order to eliminate the impact of correlative factors on the associations of plasma asprosin levels with IGR and nT2DM, multivariate logistic regression analysis was conducted and revealed that, even after controlling for age, sex, BMI, and WHR, plasma asprosin concentrations were significantly correlated with IGR (odds ratio, 120.90; 95% confidence interval, 19.52–748.65) and nT2DM (odds ratio, 20.77; 95% confidence interval, 3.74–115.35).

## 4. Discussion

Asprosin, a newly found adipokine, has been demonstrated to correlate with insulin resistance and hepatic glucose production, while its direct association with glucose dysregulation in human remains unknown. In our study, we found plasma asprosin concentrations were significantly higher in subjects with IGR and nT2DM compared with those in the NGR group, especially in IGR group. Additionally, correlation analysis demonstrated that plasma asprosin levels were significantly correlated with parameters regarding glucose metabolism, obesity, lipid profiles, insulin resistance, and *β*-cell function. However, the trends in the changes of first phase of glucose-stimulated asprosin secretion are different with that of insulin secretion among the three groups. Multivariate logistic regression analysis further confirmed the association of plasma asprosin levels with IGR and nT2DM after adjusting for age-, sex-, and obesity-related parameters.

Asprosin is a 140-amino-acid-long protein and generated by the C-terminal cleavage of profibrillin encoded by *FBN1* [5, 9, 10]. While its function remains unexplored, until recently, Romere et al. [5] revealed that the circulating asprosin is secreted by the white adipose tissue and served as a glucose sensor to regulate plasma glucose by targeting the liver under physiological condition. Beside this, they observed the circulating levels of this marker in a small population with insulin resistance. In light of its physiological function and the deep association of insulin resistance and type 2 diabetes, we asked whether the plasma asprosin is also involved with diabetes and prediabetes in human. Our study found that the plasma asprosin levels were significantly higher in subjects with IGR or nT2DM compared with those in the NGR group and were significantly correlated with nT2DM after adjusting for age, sex, and obesity parameters. Interestingly, unlike other adipokines, which rise linearly among NGR, IGR, and T2DM, our study showed the plasma asprosin levels in IGR group were the highest one among these three groups, indicating this protein might be a strong biomarker to predict prediabetes, while large-scale clinical studies are needed to further confirm this effect.

To explore the underlying mechanism, previous study demonstrated that the plasma asprosin was pathologically elevated in human and in both diet- and genetic- induced animal models of insulin resistance [5]. Consistently, our study also found a positive relationship between plasma asprosin levels and insulin resistance, indicating the asprosin-related glucose dysregulation might be through its role in insulin resistance. In addition, pancreatic *β*-cell malfunction is considered as another important mechanism in the development of T2DM [11, 12], and more and more adipokines have been reported to impact on the function, proliferation, death, and failure of *β* cell [13]. Our correlation analysis showed plasma asprosin levels were negatively correlated with indicators regarding the first-phase insulin secretion, such as AUC, AIR, and GDI and further confirmed by its negative relationship with HOMA-*β*. These results suggest that the plasma asprosin might also contribute to *β*-cell malfunction and cause the glucose intolerance. However, the pattern of first-phase glucose-stimulated asprosin secretion was not correspondent with insulin secretion, indicating the glucose regulation role of asprosin might be independent of its impact on the first-phase insulin secretion. Similarly, previous study also demonstrated a direct effect of asprosin on hepatocyte glucose production without the potential insulin compensatory effect [5]. Thus, future studies are needed to confirm its relationship and potential mechanisms involving the insulin resistance and *β*-cell function.

Inflammations are reported to participate in the pathogenesis of DM [14, 15]. Previous study revealed that asprosin induces hepatic glucose production by using cAMP as a second messenger, which was also involved in the inflammatory response [16–18]. In *FBN1* hypomorphic mice (mgR/mgR displayed a 70% decrease in circulating asprosin), proinflammatory cytokines were elevated and contributed to the formation of inflammatory diseases, such as aortic aneurysms [19]. Thus, we asked whether plasma asprosin regulated glucose metabolism through inflammation. We failed to find a correlation between plasma asprosin level and hsCRP, although the hsCRP levels rise linearly among NGR, IGR, and nT2DM groups and reached statistical significance in nT2DM patients.

The current study has some limitations that require emphasis. First, our study is cross-sectional designed and therefore causality between plasma asprosin levels and IGR or nT2DM cannot be established. Previous animal studies found that mice exposed to a single dose of recombinant asprosin showed significant increase in blood glucose levels by activating hepatic glucose production [5]. Thus, we speculated that the elevated asprosin might be a cause of glucose dysregulation, since the asprosin is an adipokine secreted by the adipose tissue, and type 2 diabetes is usually associated with the malfunction of adipose tissue which may further lead to inappropriate adipokine section. Therefore, we also cannot rule out the possibility that the elevated asprosin is the result of type 2 diabetes; further studies are summoned to clarify this. Second, as the population characteristics and environmental factors are reported to influence the secretory pattern of adipokines, our results may not apply to other populations. Third, other medicine uses which might have influence on the asprosin circulation levels are not analyzed in this study. Finally, the plasma concentration of asprosin may also be related to its catabolism, which was not assessed in our study.

In conclusion, our study demonstrated that the plasma asprosin levels were significantly higher in IGR and nT2DM subjects, especially for IGR subjects. The concentrations of plasma asprosin correlated closely with various clinical parameters of glucose and lipid metabolic disorders. Thus, the circulating asprosin might be a predictor of early diagnosis in DM and might be a potential therapeutic target for prediabetes and T2DM.

## Figures and Tables

**Table 1:** Clinical and laboratory characteristics of the study subjects.

	NGR	IGR	nT2DM
Sex (M/F)	52 (17/35)	40 (15/25)	51 (27/24)
Age (year)	54.73 ± 11.90	54.00 ± 9.62	53.73 ± 10.06
BMI (kg/m^2^)	22.76 ± 3.61	23.86 ± 3.08	24.73 ± 3.55^b^
Wc (cm)	81.08 ± 9.05	88.18 ± 6.48^b^	87.71 ± 9.36^b^
WHR	0.86 ± 0.06	0.87 ± 0.07	0.90 ± 0.06^bd^
SBP (mmHg)	120.98 ± 14.04	125.03 ± 12.76	126.75 ± 13.71^a^
DBP (mmHg)	75.42 ± 9.36	75.75 ± 9.14	79.10 ± 11.51^a^
FPG (mmol/L)	5.19 ± 0.34	6.68 ± 0.25^b^	8.27 ± 1.92^bd^
2hPG (mmol/L)	4.70 ± 0.60	6.90 ± 1.17^b^	11.79 ± 3.50^bd^
HbA1c (%/mmol/mol)	5.55 ± 0.32/37	6.01 ± 0.41/42	7.78 ± 1.89/62^bd^
FINS (mU/L)	5.92 ± 3.59	7.18 ± 3.79	8.25 ± 4.14^b^
HOMA-IR	1.38 ± 0.88	2.14 ± 1.16^b^	3.02 ± 1.64^bd^
HOMA-*β*	71.03 ± 41.79	44.83 ± 22.80^b^	38.92 ± 22.97^b^
AUC	498.95 ± 481.04	165.86 ± 109.59^b^	100.96 ± 113.99^b^
AIR	62.89 ± 61.81	17.17 ± 11.69^b^	10.93 ± 13.07^b^
GDI	1.61 ± 0.45	1.30 ± 0.31^a^	0.97 ± 0.26^bc^
TC (mmol/L)	4.41 ± 0.98	4.73 ± 1.02	4.79 ± 1.08
TG (mmol/L)	1.20 ± 0.50	1.98 ± 1.40^b^	1.90 ± 1.06^b^
HDL-C (mmol/L)	1.52 ± 0.52	1.32 ± 0.31^a^	1.27 ± 0.31^b^
LDL-C (mmol/L)	2.45 ± 0.95	2.84 ± 0.76^a^	2.83 ± 1.00^a^
hsCRP (mg/L)	1.37 ± 3.36	0.94 ± 0.78	2.31 ± 1.01^ad^
Asprosin (ng/mL)	16.22 ± 9.27	82.40 ± 91.06^b^	73.25 ± 91.69^b^

Data are presented as means ± SD. NGR: normal glucose regulation; IGR: impaired glucose regulation; nT2DM: newly diagnosed type 2 diabetes; BMI: body mass index; Wc: waist circumference; WHR: waist hip ratio; SBP: systolic blood pressure; DBP: diastolic blood pressure; FPG: fasting plasma glucose; 2hPG: 2 h postchallenge plasma glucose; FINS: fasting serum insulin; HOMA-IR: homeostasis model assessment for insulin resistance; HOMA-*β*: homeostasis model assessment for beta-cell function; AUC: area under the curve of the first-phase (0–10 min) insulin secretion; AIR: acute insulin response; GDI: glucose disposition index; TC: total cholesterol; TG: triglyceride; HDL-C: high-density lipoprotein cholesterol; LDL-C, low-density lipoprotein cholesterol; hsCRP: hypersensitive C-reactive protein. ^a^*P* < 0.05 compared with NGT; ^b^*P* < 0.01 compared with NGT; ^c^*P* < 0.05 compared with pre-DM; ^d^*P* < 0.01 compared with pre-DM.

**Table 2:** Spearman correlation coefficient of variables associated with circulating asprosin concentration in the study population.

	*r*	*P* value
Age (year)	−0.010	0.901
Sex (M/F)	−0.014	0.866
BMI (kg/m^2^)	0.097	0.249
Wc (cm)	0.185	0.027
WHR	0.002	0.983
SBP (mmHg)	0.089	0.295
DBP (mmHg)	−0.006	0.948
FPG (mmol/L)	0.466	<0.001
2hPG (mmol/L)	0.277	0.003
HbA1c(%/mmol/mol)	0.368	<0.001
TC (mmol/L)	0.017	0.840
TG (mmol/L)	0.282	0.001
HDL-C (mmol/L)	−0.143	0.088
LDL-C (mmol/L)	−0.038	0.652
FINS (mU/L)	0.131	0.120
HOMA-IR	0.29	<0.001
HOMA-*β*	−0.265	0.001
AUC	−0.478	<0.001
AIR	−0.480	<0.001
GDI	−0.568	<0.001
hsCRP (mg/L)	0.086	0.313

BMI: body mass index; Wc: waist circumference; WHR: waist hip ratio; SBP: systolic blood pressure; DBP: diastolic blood pressure; FPG: fasting plasma glucose; 2hPG; 2 h postchallenge plasma glucose; TC; total cholesterol; TG; triglyceride; HDL-C: high-density lipoprotein cholesterol; LDL-C: low-density lipoprotein cholesterol; FINS: fasting serum insulin; HOMA-IR: homeostasis model assessment for insulin resistance; HOMA-β: homeostasis model assessment for beta-cell function; AUC: area under the curve of the first-phase (0–10 min) insulin secretion; AIR: acute insulin response; GDI: glucose disposition index; hsCRP: hypersensitive C-reactive protein.
